# Climatic Conditions: Conventional and Nanotechnology-Based Methods for the Control of *Mosquito* Vectors Causing Human Health Issues

**DOI:** 10.3390/ijerph16173165

**Published:** 2019-08-30

**Authors:** Toqeer Ahmed, Muhammad Zeeshan Hyder, Irfan Liaqat, Miklas Scholz

**Affiliations:** 1 Centre for Climate Research and Development (CCRD), COMSATS University Islamabad, Park Road, Chak Shahzad, Tarlai Kalan 45550, Islamabad, Pakistan; 2 Department of Biosciences, COMSATS University Islamabad, Park Road, Chak Shahzad, Tarlai Kalan 45550, Islamabad, Pakistan; 3 Division of Water Resources Engineering, Faculty of Engineering, Lund University, P.O. Box 118, 221 00 Lund, Sweden; 4 Department of Civil Engineering Science, School of Civil Engineering and the Built Environment, University of Johannesburg, Kingsway Campus, P.O. Box 524, Aukland Park 2006, Johannesburg, South Africa; 5 Civil Engineering Research Group, School of Science, Engineering and Environment, The University of Salford, Newton Building, Salford M5 4WT, UK

**Keywords:** climate change, dengue fever, environmental management, eradication of vectors, human health, *mosquito*-borne diseases, malaria, nanotechnology-based disease control approaches, traditional disease control, public health risk

## Abstract

Climate variability is highly impacting on *mosquito*-borne diseases causing malaria and dengue fever across the globe. Seasonal variability change in temperature and rainfall patterns are impacting on human health. *Mosquitoes* cause diseases like dengue fever, yellow fever, malaria, Chikungunya, West Nile and Japanese encephalitis. According to estimations by health organizations, annually one million human deaths are caused by vector-borne diseases, and dengue fever has increased about 30-fold over the past 50 years. Similarly, over 200 million cases of malaria are being reported annually. *Mosquito*-borne diseases are sensitive to temperature, humidity and seasonal variability. Both conventional (environmental, chemical, mechanical, biological etc.) and nanotechnology-based (Liposomes, nano-suspensions and polymer-based nanoparticles) approaches are used for the eradication of Malaria and dengue fever. Now green approaches are used to eradicate *mosquitoes* to save human health without harming the environment. In this review, the impact of climatic conditions on *mosquito*-borne diseases along with conventional and nanotechnology-based approaches used for controlling malaria and dengue fever have been discussed. Important recommendations have been made for people to stay healthy.

## 1. Introduction

Vectors are the organisms responsible for the transmission of communicable diseases from faunas to human beings or among humans. The best-known vector for such diseases is the *mosquito*. Others may include flies, ticks, sand flies, triatomine bugs, fleas and aquatic snails [1]. The diseases caused by these vectors cause above one million of human deaths yearly. These diseases account for over 17% of the infectious/transmittable diseases [2]. In tropical countries, some vector-borne diseases are still ignored; e.g., lymphatic filariasis, dengue fever, Leishmaniasis, Human African Trypanosomiasis and Schistosomiasis [3]. There are about 3.9 billion people at risk of having dengue fever and 96 million cases of dengue are reported every year in 128 prevalent countries. It follows that almost half of the world’s population is at risk [4]. The prevalence of dengue fever has increased by 30-fold in the past 50 years. In 2017, the estimated malaria cases and corresponding deaths occurred were 219 million and 435,000 respectively, comprising almost 90% of sub-Saharan Africa [5]. In the South-east Asia Region, many of the vector-borne diseases are prevalent, including some *mosquito*-borne diseases such as malaria, dengue, chikungunya, Japanese encephalitis and lymphatic filariasis, snail-transmitted disease (schistosomiasis) and the sand-fly borne disease (kalaazar). Malaria is prevalent in many tropical and subtropical regions of the world excluding the Maldives, which is officially stated as being malaria-free [6].

As the globe is continuously facing climate change challenges, *mosquitoes* are benefitting from this competition. As temperatures are getting warmer, the range of disease-carrying *mosquitoes* is increasing. The increased temperature allows them to breed quicker. *Mosquitoes* may breed all-year-round in warm areas. Warm temperatures facilitate the increase in hatching and reproduction rates of *mosquitoes* [7,8]. In South-east Asia, frequent epidemics with hundreds of thousands of reported and unreported cases have arisen during the past decade, resulting in significant numbers of deaths. Countries that are more severely affected such as Pakistan, India, Bangladesh and Sri Lanka can be seen as victims of climate change in terms of *mosquito* population increases. The risk factors of these vector-borne diseases that may cause corresponding epidemics are poor sanitation, low cleanliness, climate change and the lack of efficient vector control approaches [9,10].

An increase in epidemics of Dengue is due to the absence of active gene therapy or vaccines for its control. The only reliable control approach for these diseases is their prevention and control through source reduction and insecticides. As the *mosquitoes* are developing resistance against the insecticides, the prime focus of the scientific community is to find new ways of eradicating *mosquito* populations. The purpose of this study is to assess the existing conventional and novel eradication methods and techniques, which are being used in different countries of the world to eradicate or control vectors and diseases transmitted by such vectors. Moreover, this review identifies missing gaps in the management of vectors, especially malaria and dengue fever, and approaches to manage increases in temperature due to a changing climate.

## 2. Methodology

Peer-reviewed published research papers and reports along with grey literature were selected from 1981 to 2019 (39 Years). Literature was collected from the world-renowned databases ISI Web of Knowledge (http://isiknowledge.com), Science Direct (http://www.sciencedirect.com), Scopus (https://www.scopus.com) and Google Scholar (https://scholar.google.com.pk). Relevant literature was designated on the basis of inclusion and exclusion criteria; i.e., relevance of literature to the study area on the basis of keywords used. Preference was given to the publication years 1990–2019. The search was curtained by using the following keywords: malaria; dengue fever; Chikungunya; Zika virus; Yellow fever; climate change; health impacts of *mosquito*-borne diseases; temperature and precipitation effects; conventional control methods for *mosquito*; and nanotechnology-based methods.

**Figure 1:**
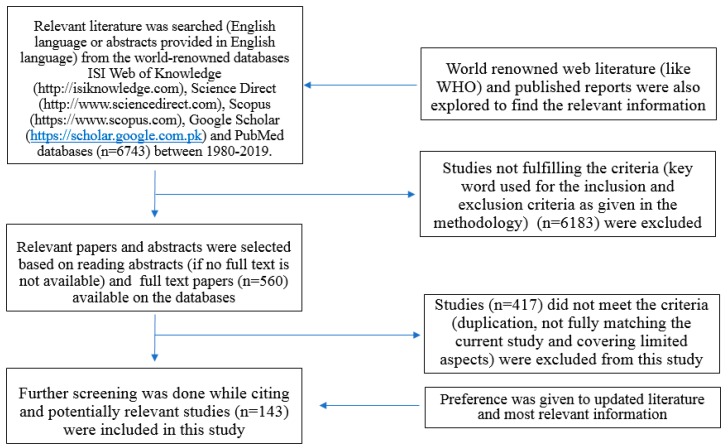
PRISMA Diagram showing the literature search strategy.

According to the exclusion criterion, literature items, which did not fulfil the above criterion were deleted (Figure 1). Literature was reviewed and properly cited to acknowledge previous studies. The author followed all steps of the methodology proposed by The Preferred Reporting Items for Systematic Reviews and Meta-Analyses (PRISMA) Group [11]. PRISMA is an evidence-based minimum set of items for reporting in systematic reviews and meta-analyses. PRISMA can be used as a basis for reporting systematic literature reviews.

## 3. Results

### 3.1. Climatic Conditions Influence on Mosquito Vectors

#### 3.1.1. Seasonality of Infectious Diseases

A high number of vector-borne diseases like dengue fever and malaria express substantial seasonal patterns around the world. Most of the insects including *mosquitoes* are poikilothermic ectotherm whose internal temperature is dependent on the ambient temperature of the environment [12]. Researchers described the relationship between water temperature and development rate that is analogous for eggs, larvae and pupae of the insects [13]. An increase in ambient temperature is linked to the increased metabolism of the *mosquitoes*. The transmission of the *mosquito*-borne diseases is at a maximum at higher temperature due to an increased rate of pupation, blood meals and a reduced extrinsic incubation period [12,14]. Similarly, optimum rainfall and precipitation is also linked to the growth and development of *mosquitoes*; and viral replication within the vector [14]. In Peru, a peak of infection with Cyclospora is seen in summer, which subsides in winter. During hot and dry seasons, other infections tend to rise, but decrease at the beginning of the rainy spell in, for example, Africa [15,16].

#### 3.1.2. Vector-Borne Diseases

There are few vector-borne pathogens in the transmission of diseases [17] that may include:

- The reproduction and survival rates of vectors in a certain environment.
- The level and degree of vector-activity; typically, the rate of biting and the period of year.
- Reproduction and development rate of pathogens inside the vector.

The reproduction and survival of pathogens, hosts and vectors is dependent on specific climatic circumstances. Any fluctuation in such conditions may affect the rate of disease transmission. Among these finest environmental conditions for vector-borne diseases, temperature and humidity are the most significant climatic factors while the airstream, daylight duration and elevation of sea level are supplementary vital considerations [18]. Table 1 shows a summary of climatic change impacts to each biotic element of vector-borne illnesses.

#### 3.1.3. Temperature Sensitivity

Extreme temperatures are mostly fatal for the survival and persistence of pathogens, but any incremental fluctuation in temperature could have varying effects. A slight rise in temperature may become deadly to the disease-causing agent, if a vector is subjected to an environmental condition such as mean temperature, which is approaching the edge of physiological tolerance for the infective agents or pathogens. On the other hand, if a vector lives in an atmosphere of comparatively lower mean temperature, an insignificant rise in temperature can accelerate the growth and replication of pathogens [19,20]. Temperature and development time are inversely correlated, and the optimum growth ranges from 22 to 32 °C [21,22].

The growth rate of a vector may also be modified by temperature due to changing their rates of biting and frequency of interaction with humans. Ultimately, a swing in temperature determines the span of transmission season [23]. Vectors can also adapt to the variations in temperature by shifting geographical distributions. The incidences of malaria in cooler environmental zones of African highlands can be the consequence of shifting habitats of *mosquitoes* to survive with augmented air temperatures [24]. Additionally, the vectors can also evolve in response to increase in temperatures. Recent evidence suggests that some vectors like the pitcher plant *mosquito* (*Wyeomyia smithii* Coquillett) has the ability to genetically adapt with climate change to survive for a longer period of time [8].

#### 3.1.4. Precipitation Sensitivity

Changeability in precipitation has straight consequences on the outbreaks of infectious diseases. Increase in precipitation elevates the incidence of vectors by growing the magnitude of current larval habitation and generating new breeding lands [8]. In addition, this increase in precipitation can also favour the growth in food materials, which ultimately supports a larger population of vertebrate reservoirs. Flooding and decrease in vector population may be triggered by unusually heavy rainfall. On the other hand, flooding may increase the probability of vector human contact. In Brazil, leptospirosis (rodent-borne disease) outbreaks have been reported after extreme flooding [25]. Unexpected droughts may cause rivers to slow in wet tropics, creating additional areas of stagnant water, which is perfect for vector-breeding sites.

#### 3.1.5. Humidity Sensitivity

The spreading of vector-borne illnesses can be greatly affected by humidity as well. The spreading of *mosquitoes*, which are also reliant on relative humidity, determines the extent of disease transmission [26]. The relative humidity is not only a limiting factor in those areas where it is more than 60%, but also temperature is considered as a main driver [27]. The survival rate of *mosquitoes* decreases in dry conditions as they can easily desiccate. Water saturation shortage as indicated by relative humidity remains the most critical factor in disease/climate models; e.g., Lyme disease models and dengue fever [23].

#### 3.1.6. Sea Level Sensitivity

At the end of the 21st century, global warming is projected to increase sea levels by 18 to 59 cm through polar ice, melting of glaciers and the thermal expansion of seawater [36]. The increase in sea level related to the change in climate results in the elimination or reduction of breeding sites for coastal wetland *mosquitoes*. Mammalian and bird hosts that reside in such environmental niches can also be endangered, which support the removal of viruses prevalent in this environment [37]. Otherwise, local invasion of salty water may turn previous freshwater wetlands into coastal ones that help to host vector species evacuated from prior coastal wetland habitats [23].

Vector-borne microbes spend much of their life cycle in cold-blooded arthropods that are subjected to various environmental influences. The transmission of vector-borne diseases affected by climatic factors includes rainfall, temperature, extreme flooding, wind and sea level rise. Flooding can dislocate and lead them to seek out food and shelter [38].

### 3.2. Mosquito-Borne Diseases and Health Impacts

#### 3.2.1. Vector-Host Relationships

It is necessary to understand the relationship between disease transmission and the connection of vectors/*mosquitoes* in the transmission of pathogens. Mechanical pathogen transmission is mainly accidental. In physically transmitted diseases, a pathogen that causes a disease needs support to move from one host to another. The arthropod obtains the pathogen from one host. The pathogen then grows in the body of the arthropod and is conveyed to another host. Within the invertebrate, the pathogen may or may not reproduce. If the parasite that causes the disease endures the sexual part of its lifecycle in a host, that host is known as the definitive or primary host (as in the case of *mosquitoes* that cause malaria). In case of malaria, a human is the transitional host in which the asexual phases of the parasite are found [39].

#### 3.2.2. Diseases Transmitted by the *Mosquito* Vectors

*Mosquitoes* act as vectors for different diseases, which transfer them from one person to another. Some of these diseases have high mortality and wide distribution. Around 28 viruses, which have a major importance in public health, are transmitted by different *mosquitoes*. Arboviruses are transmitted by *mosquito* bites. Yellow fever and Dengue fever are transmitted by *Ae.* spp., while some kinds of Encephalitis are transmitted by *Culex* spp. and by *Ae.* spp. [40,41]. Malaria is a prime disease transmitted by several species of *Anopheles*, which carry an infective form of a protozoan parasite called *Plasmodium*. It may also be transmitted by *Anophels quadrimaculatus*. *Anophels hermsi* and *Anophels freeborni* are major vectors, while *Anophels Crucians* is a minor one. Malaria is one of the serious and lethal diseases around the globe affecting millions of people mostly in tropical and semi-tropical areas.

Five species of malarial parasites (*Plasmodium falciparum*, *Plasmodium malariae*, *Plasmodium vivax*, *Plasmodium ovale*, *Plasmodium knowlesi*) are responsible for malaria in humans. Most common signs include severe headache, fever, sweating, vomiting and chills nausea. Parasites first enter the liver and then destroy red blood cells causing anemia and weakness. In complicated cases, there could be kidney and brain damage resulting in renal catastrophe, encephalitis, shock, coma and death [37].

For dengue fever, *Ae. aegypti* and *Ae. albopictus* are central vectors in human-to-human transmission. Dengue viruses are endemic in tropical areas while its highly endemic in other areas. Symptoms of dengue fever include skin rash, pain behind the eyes, severe headache, fever as well as muscle and bone pain (thus also named as break bone fever). Recovery form dengue fever is long, but death is seldom. Each of the four strains provides lifelong immunity against the virus, but exposure to the second strain may result in the dengue shock syndrome and dengue haemorrhagic fever, which are much severe than dengue [38].

The Yellow fever virus is transmitted from some forest monkeys to humans by a vector. *Ae. aegypti* and *Ae. albopictus* can transmit the virus in urban areas, while *Oc. japonicus* may also act as potential vector. The symptoms it causes closely resemble those of the dengue fever, which includes fever, backache, headache, jaundice and internal bleeding, which can lead to death [39].

The West Nile Fever is caused by the West Nile virus and transmitted via *Culex* spp. (*mosquito*). It is a type of encephalitis and may also be called arboviral encephalitis. The virus is transmitted via the *mosquito* to their main reservoir host (usually birds). Horses and humans are rather accidental hosts. The symptoms of the West Nile fever include fever, vomiting, headache and swollen lymph nodes. In complicated cases, it may involve the central nerves system [39].

This is caused by the arbovirus, which is spread through *Culex tritaeniorhynchus* (*mosquito*). The virus is transferred from pigs or birds, which are their main hosts. Like the West Nile fever, horses and humans are accidental hosts. General symptoms include headache, fever, stupor, disorientation and loss of coordination. It may also lead to paralysis, seizures, coma and death [40].

These viruses may cause encephalitis and are transferred to humans by biting of humans accidently, and thus giving a dead-end host as the vectors cannot take the virus back form the human host. It involves a transmission cycle (similar to arboviruses) from birds or small mammals, which are the primary hosts. Some people are asymptomatic, some may get mild flu-like symptoms like headache and high fever. In some of the cases, people may get encephalitis disturbing the central nervous system, which may result in paralysis, seizures, coma and death [37].

The Alpha virus, which belongs to the family Togaviridae is the causing agent of Chikungunya. It is transmitted by bites of infected *mosquitoes* to humans. *Ae. aegypti* and *Ae. albopictus* are the main agents. Symptoms associated with Chikungunya include headache, nausea, vomiting, skin rash, fever and chills. Some patients may have pain in their joints [40].

#### 3.2.3. Treatment and Vaccines for *Mosquito* Vectors

According to WHO, no specific treatment is available for dengue fever, but symptomatic treatment and the Dengvaxia^®^ vaccine are used to decrease the severity of the diseases and decrease mortality rate. The Dengvaxia^®^ (CYD-TDV) vaccine was developed in December 2015. In 2016, it was used in areas where dengue fever was highly endemic [40]. During the clinical trials, CYD-TDV was found effective in seropositive individuals. The main method to control dengue is to prevent the vector and to combat *mosquitoes*. Other vaccines developed by NIAID and Takeda are in the evaluation stage III [42]. Similarly, still no vaccines for Zika and Chikungunya virus have been found [43]. Other vaccines for Chikungunya and Zika are under clinical trials. Similarly, vaccines to provide broad protection against *mosquitoes* borne diseases like Malaria, Zika and West Nile Fever are under clinical trials by National Institute of Allergy and Infectious Diseases (NIAID) working under NIH [44]. As reported by WHO, yellow fever can be prevented by a single dose of effective vaccines, which are affordable and safe [45]. Effective drugs for malaria are easily available on the market. However, prescription is important before use.

### 3.3. Conventional Methods Used to Control Mosquito Populations

#### 3.3.1. Overview

**Figure 2:**
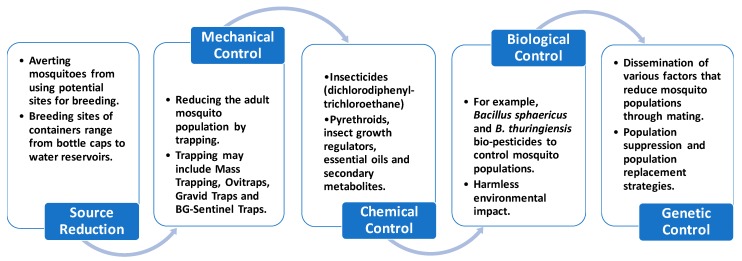
Overview of conventional methods used for the eradication of *mosquitoes*.

The purpose of monitoring *mosquito* populations is to lessen the potential for biting nuisance and disease transmission especially important for dengue and malaria. A number of effective methods are used in disease vector control and depend upon the targeted *mosquito* species. These approaches are classified into five classes: source reduction (environmental control), trapping (mechanical control), insecticides (chemical control), genetic control and biological control. An overview of conventional methods used for the eradication of *mosquitoes* is given in Figure 2.

#### 3.3.2. Source Reduction (Environmental Control)

Source reduction methods include averting *Aedes* spp. *(mosquito*) from using possible sites for breeding. These sites may consist of a varied range of containers or tanks from bottle covers to water reservoirs (Figure 2). This method is based on eradicating impermanent water tanks and closing permanent water reservoirs. This is frequently the first line of defence against *mosquitoes* such as *A. albopictus, Ae. aegypti* and *Ae. atropalpus* that favour small- and medium-sized artificial water containers and tires placed in or near homes. Source reduction also affects the dispersal of local *mosquitoes* like *Culex* spp. in the vicinity by restraining the existing sites for oviposition (process of laying eggs) [46,47,48].

Some breeding areas can be extremely fruitful for certain species. For example, the breeding sites for *A. albopictus* are catch sinks in northern Italy, ridged extension fountains in USA, and tank basins and tires in South Asia. Operative source reduction, specifically for *Aedes* spp., needs to be consistent and recurrent; e.g., washing or handling of water containers/vessels for daily use. Success depends largely on owner participation and public awareness. In a study in Brazil, a source lessening drive against *Ae. aegypti* was started using nylon nets to shelter the most productive sites such as water containers and metal barrels that resulted in a long-lasting drop in female *mosquito* populations, showing the usefulness of targeting preferable breeding sites. Regrettably, this method does not assist in tracing cryptic productive sites, which are often unseen or inaccessible such as natural reservoirs including leaf litter [49,50].

#### 3.3.3. Trapping (Mechanical Control)

Mass trapping commonly uses odor to attract *mosquitoes*. For *Aedes*, trapping methods such as ovitraps or sticky/gravid traps target gravid females. For host-seeking females, the bio-agent-sentinel traps are used. Though the usage of bioagent-sentinel traps is commonly inadequate due to the necessity for electrical supply. Ovitraps uncover the tendency of *Aedes mosquito*es to lay their eggs in small containers. An autocidal or larvicide inclusion permits the long-standing use of ovitraps with minor danger of it becoming a major source of adult *mosquitoes*. Insecticide-treated egg-laying strips are also being used [51,52].

To improve the attractivity of ovitraps, organic infusions (grass, hay and oak) can be applied. Gravid/sticky ovitraps with adhesive surfaces have also been advanced. To improve *mosquito* collections, researchers developed a bulky autocidal gravid trap with a higher release rate of water vapours and other volatile attractants [53]. As push-pull approaches, combining a repellent with some attractive incentives in tandem, are an effective control method against various *mosquitoes*. It uses the spatial repellent and contact nuisance of insecticides through insecticide-treated materials or indoor residual spraying. Still, it is important to point out that insecticide-treated materials and indoor residual spraying methods do not target exophylic *mosquitoes* such as *Ae. albopictus* [53,54]. There are some other traps like bed net traps, entry and exit traps, sentinel traps, capture-kill traps, host seeking traps, light traps and chemical based traps used for the control of *Anophels* species. Similarly, CDC traps (CO_2_ from dry ice) are used to catch 400–500% more *mosquitoes* [55,56].

#### 3.3.4. Insecticides (Chemical Control)

Chemical control of *mosquitoes* is undertaken by using natural or synthetic chemicals (insecticides). This allows for instant *mosquito* reduction control when physical and biological methods are incapable to sustain *mosquito* numbers below certain thresholds. Larvicides are the compounds proposed to be applied directly to water to control larvae populations. Adulticides along with synergists are used in obscuring and spraying adult *mosquitoes* to control their numbers.

In the twentieth century, dichlorodiphenyltrichlroethane (commonly known as DDT) was the primary artificial organic insecticide that has been used for active malaria control by decreasing the life span of gravid female *mosquitoes*. Pyrethroids and insect growth regulators are the effective compounds used worldwide in *mosquito* control approaches, as adulticides and larvicides along with ovicidal properties possessed by some insect growth regulators such as methoprene pyriproxyfen and diflubenzuron [57].

A new strategy called auto-dissemination consists of manipulating female *mosquitoes* by contamination with insecticide (pyriproxyfen) through treated nets or diffusion stations designed from modified ovitraps. The method has proved to be effective in inducing high mortality rates of *Ae. aegypti* and *Ae. albopictus* at the pupal stage. Furthermore, production and hatchability of eggs were also seen to be affected. A new approach is to synergize the sterile insect technique (SIT) with auto-dissemination to release sterile male *mosquitoes* treated with pyriproxyfen to infect females during mating [58,59,60]. Common commercially available insecticides currently used are listed in Table 2.

Plant-based insecticides received much attention, recently, due to their safe, cheap and environmentally friendly nature. Plants have a diversity of phytochemicals (used for defence) that can be applied to lessen insect outbreaks. In chemical ecology, the assessment of how specific chemicals (allelochemicals) are implicated in organism interactions with each other and with their environments is studied. These allelochemicals serve their purpose as defence mechanisms and effect molecular targets such as cellular proteins, enzymes, nervous system signal transduction (synthesis of neurotransmitter, their storage and release as well as receptor binding) and metabolic pathways in herbivores or microbes [65,66].

The secondary metabolites (phenols, alkaloids, slavanoids, sterols, terpenes, carotenoids etc.) of plants protect them against microbial parasites and arthropods; e.g., *Chloroxylon swietenia* against *Anopheles gambiae*, *Culex quinquefasciatus, Aedes aegypti* and *Calocedrus decurrens*, *Juniperus occidentalis* against adult *Ae. aegypti*, and *Eucalyptus tereticornis* against established *Anopheles stephensi*.

Plant extract-based larvicides such as essential oils, terpenes and their corresponding elements disturb biochemical processes, particularly effecting the endocrinological balance, neuromuscular fatigue, respiratory capability, neuroexcitation as well as neuro-inhibition, which results in energy depletion, oxygen deprivation, immobility and paralysis that ultimately leads to death (Table 3) [66,67,68,69].

However, it has been proposed that plant extracts, which comprise of several chemicals, are more complex in nature as compared to synthetic insecticides and, therefore, the mixture of physiological and behavioural actions of botanical insecticides prevents the development of resistance.

#### 3.3.5. Biological Control

In recent years, bio-pesticides are receiving increased interest as an effective *mosquito* control program due to their role in promoting vertebrates’ safety and lowered environmental impact. A number of organisms were discovered as potential bio-pesticides for *mosquito* control including bacteria, fungi, viruses, protozoa and fish. However, most of these organisms were not considered convenient for the development of an effective *mosquito* control program due to their limited supply. Some of the spore-forming bacteria such as *B. thuringiensis serovar israelensis* deBarjac (Bti) and *Bacillus sphaericus* Neide (Bs) were investigated against operational *Ae., Anophels* and *Cx mosquito* populations as they already showed highly toxic effects to such dipteran species. Bti bacteria spores conduct a broader range of activities against *Ae.* spp., *Culex* and *Anophels* Bs is highly specific to certain species and acts only against some *Ae.* species and majorly effect *Culex* species [81].

In *B. sphaericus* (Bs) bacteria, two insecticidal proteins, BinA with 42 kDa and BinB with 51 kDa (known as Binary toxin btx) have shown to be highly toxic for *mosquitoes*. BinA Recombinant bacteria comprising these toxins separately confirmed that BinA is extremely toxic at high dose without any need of BinB, but BinB alone does not show any toxicity. However, their synergistic effect revealed that their equal concentrations have extreme toxicity to larvae. When the *mosquito* larvae ingest btx, BinA and BinB solubilize in the midgut, where the proteases process them into 39 kDa and 43 kDa proteins. After processing, btx-active proteins are coupled to receptors present on the brush border membrane of the midgut, where the internalization of active proteins occurs leading to the cell lysis, and ultimately, lysis and inflammation of midgut, which results in the insect’s death. Another *mosquito*cidal toxin Mtx (100 kDa) appears to be highly toxic in protease negative Bs strains that has been synthesized in low toxicity strains [82,83,84,85,86,87,88].

In Bti bacteria, two insecticidal protein groups Cyt (cytolysins) and Cry (crystal delta-endotoxins) were also shown to be effective for *mosquito* control [50]. Bti toxins contain four polypeptides: Cry IVA (125 kDa), Cry IVB (135 kDa), Cry IVD (68 kDa) and Cyt A (28 kDa). They are assembled into crystals during sporulation. The genes encoding these Cry toxin proteins are present on the plasmid of the bacterium [89,90]. Cry proteins bind to definite receptors present on the midgut epithelium of the insect, while Cyt proteins do not distinguish specific sites present in the midgut. After ingestion, Bti crystals are processed into smaller fragments by proteases. The activated fragments interrupt the osmotic equilibrium of epithelium cells through making pores into the membrane causing cell death. Most of the *mosquitoes* die within hours due to a paralyzed gut [91,92,93].

However, there are some resistance problems with these insecticides, because of the insects’ increased evolutionary rate. The factors linked to an increased evolutionary rate are elevated productive rates of species, increased and larger number of progenies, shorter generation times, and higher genetic variability among local species [94].

Recently, genetic engineering techniques are being used for improving bacterial larvicides for *mosquito* control. The recombinant bacteria of Bti and Bs strains are ten times more effective than wild strains that are also being used for larger scale larvicides production. The recombinant strains comprise all the endotoxins of Bti (Cry4A, Cry4B, Cry11A, and Cyt1A) along with Bs (Binary toxin). The synergy of these toxins will eradicate *mosquito* populations more effectively [95,96].

#### 3.3.6. Genetic Control

Genetic approaches are attaining greater attention as alternatives to conventional methods for *mosquito* control. Dissemination of various factors that reduce *mosquito* damage by mating is defined as genetic control. Such heritable factors are introduced into target species so that the modified *mosquito* acts as biocontrol agent and their dependence on vertical capacity distinguishes them from biological control methods. Genetic strategies are classified based on their intended outcome into population suppression, population replacement and other key aspects of genetic element persistence [96].

Population suppression programs decrease the number of vectors in the target species, which is comparable to the purpose of insecticide-based strategies. For example, the sterile-male program is intended to suppress the target population when sufficient numbers of sterile males are released. These modified sterile males are disseminated in the environment to mate with unmodified females resulting in the death of the progeny of this mating, and can potentially lead to population eradication [87]. Additionally, population replacement methods diminish the midgut transmission capability of nearly all of the *mosquitoes*’ present in the target population. For example, a new character (trait) like lower capability to spread a pathogen into the specific *mosquito* population will be advantageous for humans, while probably being lethal for the *mosquito* population [97,98].

Persistence or invasiveness is the ability to which the alteration (trait) will be present or disperse in the target group after its release. Concerning self-limiting strategy, the trait has the tendency to vanish from the target *mosquito* population until replaced by the release of further altered *mosquitoes*’ periodically. In self-sustaining strategy (gene drive system), use of the proposed alteration can persevere forever and possibly even disperse inside the primary target species or to other species [99].

Regarding the self-limiting strategy, as through the use of sterile male systems, the mutations or inheritable lethal genes are introduced where only a females’ offspring is affected [100]. Sterile insect techniques (SIT), where DNA-damaging agents like γ-radiations are used to introduce lethal mutations and nucleases in the germ line, are expressed to cause chromosomal breaks [101]. Another approach, release of insects carrying a dominant lethal (RIDL) genetic system can be applied to induce toxic gene expression in zygote relative to the father providing more flexibility (to act at a certain time in development), but at the same time make this strategy more vulnerable to the environment [102].

Sterility can also be introduced by diverse strains of *Wolbachia* bacteria by artificial infection. *Wolbachia* is maternally transmitted through the mother to her offspring. Female-killing systems can also offer “sterilization”, where released male offspring are homozygous for a female-specific lethal gene that will result in inheritance of the female lethal gene copy; as an effect, offspring (daughters) would die. This approach is known as female-specific RIDL (fsRIDL). It provides *mosquito* population control much like classical SIT [102,103].

In self-sustaining systems, gene drive is the common phenomenon where all selfish-DNA constituents are introduced that can persist over a target population without giving a suitable benefit to the *mosquitoes*’ carrying them. Many selfish-DNA elements are known that are being used such as invasive *Wolbachia, transposons*, synthetic *Medea-*like elements and the Homing Endonuclease Gene (HEG) [104,105]. The HEG is expressed either as separate genes within introns, as fusions with host proteins or lethal gene where they catalyse the genomic DNA hydrolysis within the cells that produce them.

Artificial *Medea*-like elements, originating from the naturally present selfish *Medea* elements of *Tribolium castaneum* have been created. The method uses reasonably intended synthetic elements that have the ability to replicate the inheritance pattern of *Medea* deprived of deriving its molecular components [106]. Furthermore, *Wolbachia* strains are selfish-DNA elements, which can spread by target *mosquito* species through altering their host’s reproductive biology (generating CI to kill uninfected females).

Gene drive is another technology to propagate a particular gene by altering the probability from the natural to transmit a specific allele in the next generation. This can be performed in different ways to control *mosquito* species. However, a detailed discussion is beyond the scope of this review.

#### 3.3.7. Integrative Vector Management Approach

This approach is used to achieve globally-set targets to control vector-borne diseases. This includes situation analysis, monitoring and evaluation, surveillance and capacity building. The overall purpose is to apply all strategies to control larvae, adults and bites from *mosquitoes*. The overall use of biological, chemical and physical methods for the control of *mosquitoes* is encouraged. Integrated practices are more advisable and a sustainable approach for lowering the *mosquito* population is advantageous. Appropriate ecofriendly plans and strategies should be adopted [8].

## 4. Nanotechnology-Based Methods

### 4.1. Overview

Nanotechnology-based methods have revolutionized a varied area of research fields including entomology. Nanotechnology has a good potential to combat climate change. There is a possibility to develop nanomaterials in the environment naturally. Temperature change can influence the reaction rate and disturb the formation and synthesis of nanoparticles [107]. Nanoparticles show a high antimicrobial activity against various microbes, because of their large surface to volume ratio that is responsible for their high catalytic activity. Nanoparticles have been testified as valuable when compared to chemical insecticides targeting adult *mosquitoes* and young larval instars. This is likely due to the fact that the overuse of insecticides has led to unfavorable consequences on the environment, plants and human health, along with the insecticide resistance development in targeted populations. Currently, a number of nanomaterials are being used to control *mosquito* vector-borne diseases.

### 4.2. Nanoliposomes

Liposomes are synthetic nano-assemblies up to few hundred nanometers in diameter comprising one or more outer phospholipid bilayers enclosed by an aqueous core. As a nano-carrier, both hydrophobic and hydrophilic particles can be captured within aqueous core and outer lipophilic layers, respectively. Nanoliposomes are also immunologically compatible due to their targeted uptake by cells through surface adjuvants such as antigens, antibodies and cell-associated specific ligands [108,109]. Nanoliposomes have been already used for malarial treatment as well as for the delivery of malaria vaccine. At present, efficient malarial therapy is restricted due to their deadly side effects and the potential for development of drug resistance. However, the encapsulation of a therapeutic drug (chloroquine) within nanoliposomes can effectively adjust the bioavailability and drug dose within the body that significantly lessen the drug toxic effects, and the risk of drug resistance [110].

Currently, innovative gel-core nanoliposomes that use a composite of polymer and lipid-based carrier systems have been designed and confirmed for the controlled delivery of Pfs25 along with CpGODN (immune-stimulatory adjuvant) as malarial vaccine. In gel-core nanoliposomes, the integration of a polymer into the interior aqueous part of the liposome increases its stability, permitting slower drug release. The slow diffusion through the polymer gel core and phospholipid bilayers actively controls the drug release rate, which enables the capability of nanoliposomes for lasting antigen persistence without the need of boosting [111].

### 4.3. Nanosuspensions

The biphasic approach containing drug particles of submicron-sized dissolved in an aqueous solution is defined as nano-suspensions, prepared through top-down and bottom-up techniques to lower the size of particles. The purpose of using nano-suspensions is that their lower size increases their surface area, providing higher bioavailability of the drug and lowers its toxicity [112].

The anti-malarial drug Lumefantrine is used to treat multi-drug resistant malaria by inhibiting the ability of *P. falciparum* to convert heme into toxic hemozoin. The increased accumulation of toxic heme in a parasite causes its death and stops the malarial infection. However, Lumefantrine has lower water solubility that can lead to impaired bioavailability through oral or dietary route [113,114]. Nano-suspensions based drug delivery can be useful to combat malarial infections. Improved antimalarial activity of lumefantrine-based nano-suspensions by reducing its size from 72 μm to 0.251 μm through in-vitro and in-vivo experiments against *P. Yoelii nigeriensis* and *P. falciparum* has been observed [115].

The antimalarial action of the dihydroartemisinin (DHA) drug against *P. falciparum* in in-vitro trials has been demonstrated. The lower concentration of drugs in nanosuspensions have decreased side effects other than antimalarial activity [108].

### 4.4. Polymer-Based Nanoparticles

Polymer-based nanoparticles are biodegradable and biocompatible solid-colloidal particles ranging from 1 to 1000 nm in size. The second most plentiful polysaccharide is chitin-derived. Chitosan nanoparticles have many advantages owing to their increased stability, biodegradability, biocompatibility, water solubility, controlled drug release, lower toxicity, non-immunogenic and ease-of-fabrication methods, which make them ideal candidates for *mosquito* control [116,117].

Primaquine (PQ) is a commonly used anti-malarial drug that opposes the relapsing form of malaria by *P. vivax* and *P. ovale* plasmodium parasites. However, PQ has some adverse effects due to non-specific targeting, its lower bioavailability and short half-life. Reformulation of PQ into chitosan-based nanoparticles can be a promising approach to overwhelm these side-effects. Scientists tested PQ-loaded solid lipid nanoparticles that showed effective vector reduction with increased half-life, reduced side effects and increased bioavailability compared to the conventional PQ drug [115,116,117,118].

Chitin is a major component of all insects including *mosquitoes*. It is part of their exoskeleton cuticle and other tissues like foregut, midgut, hindgut and trachea. Chitin biosynthesis is entirely dependent on the chitin synthases enzymes by catalysing the transmission of sugar from triggered sugar donors to definite acceptors present in *mosquitoes*. CHS-A is a chitin synthesis gene that is present in the cuticle and foregut, hindgut and trachea tissues, while the chitin synthesis gene CHS-B is specifically linked to the midgut. Epithelial cell’s chitin biosynthesis is associated with the peritrophic matrix (PM) [119,120,121].

RNA interference (RNAi) technology is an eco-friendly targeted approach to control *mosquito* vectors through small interfering RNA (siRNA) activated post-transcriptional gene silencing [114]. Recently, scientists have developed chitosan/dsRNA-based nanoparticles against the African *mosquito Anopheles gambiae* to silence the chitin synthase genes CHS-A and CHS-B [117].

*Mosquito* growth and development mostly depends on chitin synthases. Consequently, chitin synthase genes are ideal targets to eradicate *mosquito* vector populations. Other polymeric nanoparticles such as poly glycolic acid (PGA), poly d, l-lactic acid (PLA), poly d, l, -lactic-co- glycolic acid (PLGA) and poly Ɛ-caprolactone (PCL) are also being used to encapsulate drug active-compounds due to their increased drug stability, control drug release and lower toxicity [122,123].

The primaquine (PQ) drug can be encapsulated in polymeric nanoparticles to control malarial parasite infection [124,125]. Reserachers synthesized nano-chloroquine (Nch) particles from 150–300 nm. Nch particles were tested against liver and spleen resulting in ~29% and ~37% reductions in liver and spleen damage, respectively, compared to conventional chloroquine [126].

### 4.5. Green-Synthesized Metallic Nanoparticles

The misuse of chemical insecticides is linked to major concerns regarding their toxicity and ecological problems, but green synthesized nanoparticles have reported to be advantageous, because they are eco-friendly and cost-effective compared to conventional methods. For an effective vector control approach, appropriate formulations of botanical-based insecticides need to be improved in terms of their persistence; green synthesized nanoparticles with suitable reducing and capping agents are employed to attain their optimal activity with reduced size and different shape. There are several biophysical techniques such as UV-vis spectroscopy for nano-synthesis monitoring over time, Fourier Transformed Infrared Spectroscopy (FTIR) for functional group studies involved in capping and reduction, Energy Dispersive X-ray (EDX) for elemental composition analysis and X-ray diffraction (XRD) spectroscopy for analysing crystalline structures of nanoparticles [127].

The efficacy of green-synthesized Ag nanoparticles obtained from Dicranopteris linearis, Chenopodium ambrosioides, Aristolochia indica, Gracilaria edulis, Couroupita guianensis and Phyllanthus niruri plant extracts was validated against Anopheline aedine and Culicinae spp. *mosquito* vectors. The LC_50_ values of these nano-larvicides was in the range of 1 to 30 μg/mL and the complete eradication of these larval species was recorded within 72 h after first treatment [128,129].

Egg hatchability of *Ae. aegypti* and *Culex Quinquefasciatus* was 100% decreased after exposure to green-synthesized Ag nanoparticles (30 μg/mL) from *Sargassum muticum.* Ag-nanoparticles from *D. linearis* and *S. maritima* also reduced the *Ae. aegypti* egg hatchability after their treatment with 25 μg/mL and 20 μg/mL concentrations, respectively. The *Culex Quinquefasciatus* eggs were resistant to larvicidal effect of green-synthesized nanoparticles in comparison to *Anophels stephensi* and *Ae. aegypti.* Lately, *Rubus ellipticus*-synthesized Ag nanoparticles showed no hatchability of *Ae. aegypti, Anophels stephensi* and *Culex Quinquefasciatus* with single dosage of 75 μg/mL, 90 μg/mL and 60 μg/mL, respectively [130].

Similarly, a number of leaf extract-based Ag nanoparticles have shown their adulticidal activity against *Ae. albopictus, Ae. Aegypti, Anophels stephensi and Culex Quinquefasciatus.* Unfortunately, no possible toxicity mechanism of ovicidal and adulticidal green-nanoparticles is known, yet [128]. However, it has been assumed that nanoparticle toxicity against *mosquito* young larvae is because of their ability to penetrate into exoskeleton and their binding to DNA phosphate groups and sulphur-rich proteins. Consequently, this results in DNA alterations, rapid protein degradation and a decrease in membrane permeability, which causes loss in cell function and cell death [129,130,131].

Recently, some studies reported that nanoparticle exposure to *Ae. Aegypti* and *Anophels Stephensi* results in adult longevity and female fecundity. *Hypnea musciformis*-fabricated Ag nanoparticles reduced the longevity of *Ae. Aegypti* males from 15.64 to 5.32 days and *Ae. Aegypti* female longevity from 26.87 to 17.34 days. Similarly, exposure of these nanoparticles to *Ae. Aegypti* results in the reduction of female fecundity from 150 eggs to 98 eggs after 50 ppm dosage.

*P. aquilinum*-fabricated Ag nanoparticles (50 ppm) reduced the longevity of *Anophels Stephensi* males and females from 21.44 to 9.90 days and from 37.18 to 20.90 days, respectively. While, post-treatment of 50 ppm nanoparticles reduced the *Anophels Stephensi* female fecundity from 145 to 66 eggs [132,133]. This method might indicate a way to control *mosquito*-borne diseases more effectively.

## 5. Drive to Transform Sick to Healthy Life under Changing Climatic Conditions

*Mosquitoes* are likely to benefit from climate change [8]. Apart from suitable *mosquito* breeding sites, temperature, precipitation and absolute humidity of more than 60% can affect parasite transmission in malaria. As the globe is continuously warming, more incidences of dengue and malaria as well as an increase in the distribution of *mosquitoes* have been related to climate change. The range of disease-vectors may not only increase in latitude but also in altitude. As the *mosquito* population is increasing globally with time, they will colonize new suitable areas where the temperature is warmer fulfilling their needs.

Due to warmer temperatures, the cell metabolism of *mosquitoes* will also increase as chemical reactions are increasing. In warm temperatures, mature females feed more frequently and digest blood rapidly, thus producing more progeny. Hard-freeze areas in the winter season have less *mosquitoes* as they cannot breed during this time and usually eggs are destroyed. However, some species are possibly able to live during the winter season, if the temperature is not too low. At higher temperatures, *mosquitoes* hatch at a higher rate, subsequently their rate of development and reproduction will rise exponentially [123], and the range of *mosquitoes*-borne diseases will increase as well [6].

As *mosquitoes* are poikilothermal, the development of parasites in the *mosquito* is reliant on the external temperature. For *P. vivax* and *P. falciparum*, the nominal temperature at which the development of parasites can be accomplished is 16.5 °C and 14.5 °C, respectively. Higher temperatures accelerate their development, because sporozoites are formed quickly. It follows that the life cycle is completed sooner. Therefore, the likelihood of malarial infection will increase with higher temperatures. There is an inverse relationship between the longevity of *mosquitoes* and temperature. At higher temperatures, *mosquitoes* and their parasites die. There is an optimum temperature range for Plasmodium transmission (22–28 °C for *P. vivax* and *A. messeae*, and 26–32 °C for *P. falciparum* and *A. gambiae*) [134].

**Figure 3:**
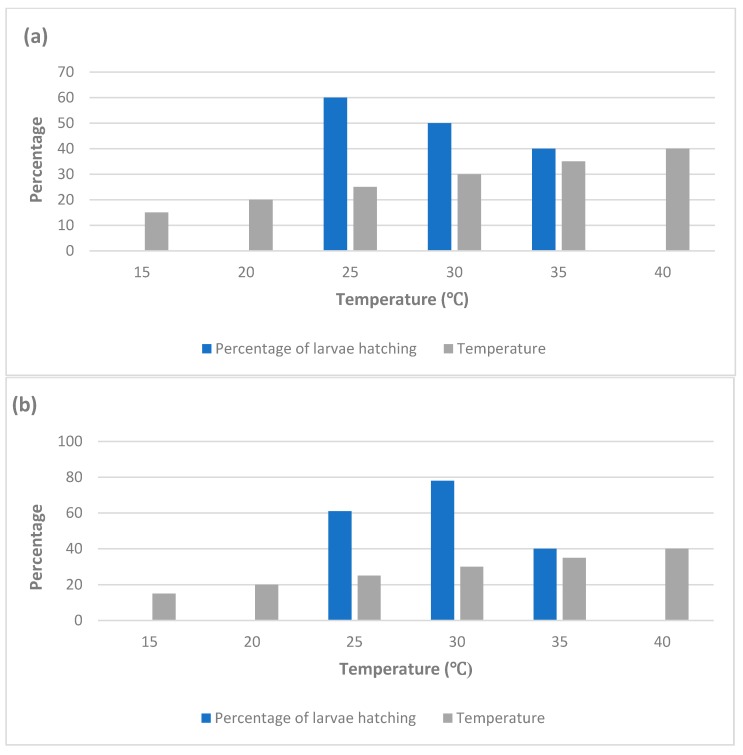
Fertility rates of eggs sustained at different temperatures and humidity values: (**a**) relative humidity (rh) of 60%; and (**b**) relative humidity (rh) of 80%; n = number of eggs (adopted from [135]).

The number of females that laid eggs and the number of eggs laid are influenced by both temperature and humidity (Figure 3). A reduction in oviposition rate was detected with increase in temperature, while the intensity of the reduction was affected by humidity. At high temperature (35 °C) and low humidity (relative humidity of 60%), a low oviposition rate (55 ± 4.8 eggs) was observed for adult females. On the other side, at a low temperature (25 °C) and high humidity (relative humidity of 80%), a high oviposition rate (99 ± 3.6 eggs) was noted (Table 4) [135].

A high humidity is suitable even up to saturation (80% or more of relative humidity) at 28 °C. At comparatively lower temperatures (18–20 °C), life expectation may be very substantial even at low humidity and *mosquitoes* can be kept alive if given water only (without any food) for several weeks. At a temperature of 25 °C and relative humidity of 70% with water provided, low mortality was observed among females earlier the seventh day either fed or not. The duration of life under such conditions without food is about 6 to 8 days. A maximum of 12 days of life expectancy was observed. However, at relatively high temperatures (28 °C) and in the absence of high humidity, a considerable mortality of males was observed even soon after emergence. After the seventh day of emergence, mortality among unfed males and females increased quickly and a high mortality by the tenth day was noted. Few *mosquitoes* were alive at the fourteenth day at 25 °C even at high humidity and with water given [123,135,136,137].

A temperature below freezing appeared to be fatal for the adults, if continued for 24 h. At freezing temperature, the *mosquitoes* are completely immobilized. At 4 °C for 1 h, all *mosquitoes* survived, but extended exposures at 4 °C killed them. However, between 7 and 9 °C, *Ae. aegypti* *mosquitoes* were alive for about 82 days. Moreover, half of the females were alive after 30 days. The optimal temperature for *mosquitoes* is usually around 28 °C. An increase in temperature would be progressively disadvantageous for survival. More than 40 °C is lethal for *mosquitoes* [138,139].

In order to address climate change, there are two common strategies: mitigation and adaptation. These approaches are interrelated with each other, but the basic difference is that adaptation is linked to the response to the fluctuating climate changing the lifestyle in which humans live as a society, while mitigation means restraining climate change, mainly by lowering greenhouse gas emissions.

Scientists are working to improve the efficacy and availability of alternative energy approaches. To increase domestic energy efficiency, simple measures such as to drive less by biking and use of public transportation would play important roles in reducing climate change worldwide. To limit climate change, our priority should be on changing our own behaviour by replacing to energy sources that release greenhouse gases [140]. Alternative approaches to reduce emissions and lowering temperature can decrease the impacts on human health. Furthermore, adaptation to a changing climate is also important. There is a need for thoughtful planning to minimize hazards.

## 6. Pakistani Perspective with Reference to Other Countries

Climate change contributes to the spreading of diseases. Conferring to the World Health Organization (WHO), the most fatal vector-borne malarial disease claimed 627,000 lives worldwide in 2012. However, a fast-growing vector-borne disease is dengue; up to 30-fold rise in disease occurrence during the last 50 years [4,5].

Vector-borne diseases add to most health-related complications in developing countries. Since 2005/6, Pakistan is facing steady epidemics of dengue and dengue haemorrhagic fever (DHF). Since 2010, Pakistan has been severely impacted by dengue fever, which led to 16,580 confirmed cases and 257 deaths in Lahore alone. Almost 5,000 confirmed cases and 60 deaths in the other parts of the country have been reported. The entire country is susceptible to dengue through the changeable trends in climate. Pakistan has been categorized in “group three” (Eastern Mediterranean Region) by the World Health Organization together with countries such as Afghanistan, Djibouti and Somalia due to the current alarming situation of vector-borne diseases with an estimated impact of 1.5 million cases annually [7,140]. Worldwide statistical data on dengue cases and their mortality are shown in Table 5 [141].

The underprivileged poor in exposed societies existing in distant rural areas with restricted access to health amenities suffer the most. Inappropriate settlements in slums that are without any satisfactory water and sanitation systems are also a challenge. Due to poor living environments, vector-borne diseases flourish. Increasing temperatures and humidity levels are likely to elevate vector-borne disease transmissions such as dengue, malaria, yellow fever and encephalitis. Different studies concluded that an increase in average temperature will enhance the reproduction cycle of the dengue fever virus.

**Figure 4:**
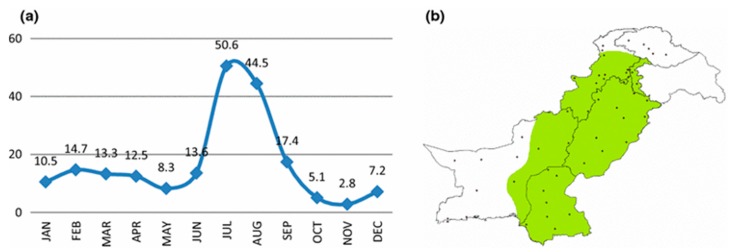
(**a**) Monthly area weighted rainfall (mm) for Pakistan; and (b) locations of meteorological stations that are available for climate data. Note that the *green color* shows the monsoon zone (adopted from [143]).

Changing climatic conditions in terms of temperature, rainfall and humidity as well as low levels of immunity among poor populations enhance malaria outbreaks. A temperature range between 20 and 30 °C, a humidity level above 55% and the presence of breeding sites in standing water to rainfall are optimal parameters for development, longevity and transmission of malaria. In Pakistan, vector-borne diseases (predominantly dengue fever and malaria) proliferate after the monsoon. From July to September, more than 80% of total vector-borne diseases are reported every year. The highest numbers of incidences have been reported for the Punjab and followed by Balochistan, Federally Administered Tribal Areas and Khyber Pakhtunkhwa in this order [142]. The monthly rainfall (mm) in Pakistan and locations of meteorological stations are shown in Figure 4. The indicated monsoon zone corresponds to the most affected areas [143].

## 7. Conclusions 

To fight drug and insecticide resistance of *mosquitoes* developed through conventional treatment methods to prevent dengue and malaria outbreaks, nano-based technology that is considered to be health- and eco-friendly such as food grade nanoparticles can be used. Food grade nanoparticles have no side effects compared to metallic nanoparticles. They are both eco- and human-friendly. In order to overcome complications, transgenic *mosquitoes* (genetically modified) and a combination of drugs have been tested. However, these methods are challenging, because *mosquito* trans-genes are destructed, and multi-drugs can be found in their bodies. Nanomedicines can be anticipated and might aid treatment for malaria and dengue fever. A multi-drug combination employing nanoparticles could be promising. Specific consideration should be given to metallic like Zno nanoparticles, food grade nanoparticles like Curcumin and biodegradable nanoparticles with multi-drug encapsulation to slow down the evolutionary progression. Especially food-based nanoparticles, which have low toxicity compared to metallic drugs, multi-drugs and bio-drugs, might help to eradicate vector-borne diseases in developing countries such as Pakistan in the future. Further investigations are required to test the larvicidal activity of food-based nanoparticles against the larvae of *mosquitoes* causing malaria and dengue fever. 

### Outlook

The following recommendations are made to people to stay healthy:

- An environmental management approach is very important for the control of breeding sites of *mosquitoes* especially during and after monsoons. These sites include open drainage, marshes and standing water in pots.
- Control of breeding sites as a result of increased rainfall and change in rainfall pattern due to climate change, which increases the vector population like *mosquitoes* and provide more breeding spaces.
- Awareness campaign among the general public at community level and in schools regarding the spread and control of malaria and dengue fever (especially the time period for the diseases).
- Personal protective approaches include use of proper clothing, nets, *mosquito* repellent lotions and other protective measures in homes, businesses, schools and offices.
- Allocation of enough funds by the authorities for health departments to control the spread of malaria and dengue fever in the community.
- Use of biological, chemical and physical methods to diminish *mosquito*-related diseases.

## Figures and Tables

**Table 1:** Climate change impacts on vector-borne diseases [23].

Impacts of Temperature on Certain Vectors and Vector-Borne Pathogens	Impacts of Changes in Precipitation on Vector-Borne Pathogens	Impacts of Higher Sea Level on Vector-Borne Pathogens
*Vector* Vector survival can increase or decrease depending on species [8]. Some vectors have an increased survival rate at higher temperatures, higher altitudes and latitudes [28]. Vector susceptibility to some pathogens varies (e.g., an increase in temperature lessens the size and lowers the activity of some vectors) [29]. Vector population’s growth rate can be changed [30]. Fluctuations in rate of feeding and host contact can change the survival rate. Alteration in population’s seasonality [31].	*Vector* Larval habitat and population size may increase due to the increased rain by providing new habitat. Snowpack and excess rain can eradicate habitat due to flooding (declining vector population). Lower rainfall can provide habitat by triggering rivers to dry into pools, resulting in dry season malaria. Lower rainfall can lead to a rise of container-breeding *mosquitoes* by imposed increased water storage. [27,32] Longer rainfall events can coordinate vector host-seeking and transmission of virus. Increased humidity can influence vector survival. [33]	*Vector* Reducing or eradicating breeding sites of *mosquitoes* (e.g., *Cs. melanura*). The relationship between vector biology and climatic conditions is complex due to the natural capability of vectors to seek out appropriate microclimates for their survival such as hiding places under vegetation, pit toilets during hot or dry weather and tunnels during cold weather [23].
*Pathogen* At higher temperatures, decrease in extrinsic incubation period of pathogens [34]. Variations in transmission season. Variations in distribution. Lower viral replication [29].	*Pathogen* Some direct effects but humidity significantly impacts on the malaria-causing parasite growth in the host (anopheline *mosquito*) [35].	
	*Vertebrate host* High rainfall can influence vegetation, food accessibility. and size of population. Elevated rainfall can cause flooding, which leads to a reduced population size but increases human contact.	

**Table 2:** Commonly used pesticides on the market against vectors.

Name	International Union of Pure and Applied Chemistry Name	Molar Mass	Pack Size	Applications	Reference
Fendona 10% [C_22_H_19_C_l2_NO_3_]	[cyano-(3-phenoxyphenyl) methyl] 3-(2,2-dichloroethenyl)-2,2-dimethylcyclopropane-1-carboxylate	416.300 g/mol	100, 200 and 1000 mL	It has a broad action spectrum against a large variety of insect pests in households and public facilities.	[61]
Lambda Cyhalothrin [C_23_H_19_ClF_3_NO_3_]	cyano-(3-phenoxyphenyl) methyl] (1R,3R)-3-[(Z)-2-chloro-3,3,3-trifluoroprop-1-enyl]-2,2 dimethylcyclopropane-1-carboxylate	449.854 g/mol	250 and 500 mL	Targets aphids, butterfly larvae, cockroaches, *mosquitoes* and flies.	[62]
Deltamethrin [C_22_H_19_Br_2_NO_3_]	Cyano (3-phenoxy-phenyl) methyl; 3-(2,2dibromoethenyl)-2,2-dimethylcyclopropanecarboxylate (CA); [partial diff]-cyano-m-phenoxy benzyl, (1R,3R)-3-(2,2-dibromovinyl)-2,2-dimethyl -cyclopropanol-carboxylate, (S)-[partial diff]-cyano-3-phenoxybenzyl (1R)-cis-3-(2,2-dibromovinyl)-2,2-dimethylcyclopropane-carboxylate	505.240 g/mol	25 g/L	Control of the malaria vector as a stomach poison for insects.	[63]
Fenpropathrin [C_22_H_23_NO_3_]	2,2,3,3-Tetramethylcyclopropane carboxylic acid cyano(3-phenoxyphenyl) methyl ester	349.42 g/mol	1000 L (mini-mum order)	Controls a variety of pests; specifically, mites present in fruits and vegetables.	[64]

**Table 3:** Plant-extracted essential oils and their mechanisms of action (after [65]).

System	Active Compound	Mechanism	Plant	References
**Cholinergic system**	Essential oils	Acetylecholinestrase (AChE) inhibition	*Azadirachtina indica*, *Lavendula* spp. and *Mentha* spp.	[70]
	Veratrin	Nerves sodium channels	*Schoenocaulon officinale*	[71]
	Nicotine	Cholinergic acetylcholine nicotinic receptor agonist/antagonist	*Nicotiana* spp., *Haloxylon salicornicum*, *Delphinium* spp. and *Stemona japonicum*	[72]
**GABA system**	Thymol and Silphinenes	GABA-gated chloride channel	*Thymus vulgaris*	[73]
**Mitochondrial system**	Pyrethrin	Potassium ion and sodium exchange disturbance	*Crysanthemum cinerariaefolium*	[74]
	Rotenone	Cellular respiration inhibitor (mitochondrial complex I electron transportinhibitor or METI)	*Lonchocarpus* spp.	[75]
	Ryanodine	Calcium channel disturbance	*Ryania* spp.	[76]
	Sabadilla	Alter nerve cell membrane action	*Schoenocaulon officinale*	[77]
**Octopaminergic system**	Essential oils	Octopaminergic receptors	*Cedrus* spp., *Pinus* spp., *Citronella* spp. and *Eucalyptus* spp.	[78,79]
	Thymol	Block octopamine receptors by functioning through tyramine receptors cascade	*Thymus vulgaris*	[78,80]
**Miscellaneous**	Azadirachtin	Anti-mitotic (G2/M Phase)	*Azadiractina indica*	[71]

**Table 4:** Mean number of eggs laid by *Aedes aegypti* females at different temperature and humidity values (after [135]).

Temperature	Relative Humidity of 60%		Relative Humidity of 80%	
	Eggs (Sample Number)	Oviposition Variation	Eggs (Sample Number)	Oviposition Variation
**25 °C**	85.99 ± 3.16 (102)	4–160 (37.25%)	99.08 ± 3.56 (92)	4–155 (55.43%)
**30 °C**	82.89 ± 3.33 (111)	2–143 (37.84%)	75.75 ± 5.03 (75)	1–144 (45.33%)
**35 °C**	54.53 ± 4.81 (55)	1–126 (14.55%)	59.62 ± 3.41 (79)	2–132 (7.59%)
**Total**	78.25 ± 2.2 (268)		79.29 ± 2.53 (246)	

**Table 5:** Statistical data on dengue cases and the corresponding mortality during the period between 2009 and 2017.

Reports	Saudi Arabia	Sri Lanka	Pakistan	Nepal	Malaysia	India	China	Bangladesh
Reported cases	1600	35,008	1085	30	41,486	17,000	322	510
Mortality	N/A	346	13	0	90	105	0	0
Reported cases	2200	34,188	11,024	917	46,171	28,000	260	405
Mortality	N/A	229	40	5	135	112	0	0
Reported cases	2400	28,473	17,057	79	19,884	19,000	160	1310
Mortality	N/A	246	219	0	38	171	0	8
Reported cases	800	44,461	639 (Karachi only)	183	21,900	50,000	610	750
Mortality	N/A	220	4	0	37	240	0	1
Reported cases	4200	32,063	4388	642	43,346	70,000	4779	1790
Mortality	N/A	N/A	32	0	95	140	0	3
Reported cases	1950	47,502	1400	355	108,698	40,571	47,056	355
Mortality	N/A	N/A	16	0	215	137	6	0
Reported cases	4200	29,777	3900	1500	120,836	99,913	4230	3162
Mortality	N/A	N/A	11	0	336	220	0	N/A
Reported cases	N/A	55,150	2500	594 till October	100,028	1,29,166	2098	6010
Mortality	N/A	N/A	3	N/A	231	245	N/A	N/A
Reported cases	N/A	185,688	N/A	854 till October	82,840	1,53,635	3195	N/A
Mortality	N/A	N/A	N/A	N/A	171	226	N/A	N/A
